# The implications of non-anatomical positioning of a meniscus prosthesis on predicted human knee joint biomechanics

**DOI:** 10.1007/s11517-020-02158-0

**Published:** 2020-04-11

**Authors:** Hamid Naghibi, Dennis Janssen, Ton van den Boogaard, Tony van Tienen, Nico Verdonschot

**Affiliations:** 1 Robotics and Mechatronics Lab, Technical Medical (TechMed) Centre, University of Twente, Building Carré, Room CR 3607, P.O. Box 217, 7500 AE Enschede, The Netherlands; 2 Radboud Institute for Health Sciences, Orthopaedic Research Lab, Radboud University Medical Center, 6525 GA Nijmegen, The Netherlands; 3 Nonlinear Solid Mechanics, Faculty of Engineering Technology, University of Twente, 7522 NB Enschede, The Netherlands; 4 Laboratory of Biomechanical Engineering, University of Twente, 7522 NB Enschede, The Netherlands

**Keywords:** Meniscus injury, Meniscus prosthesis, Implant positioning, Finite element analysis, Gait simulation, Osteoarthritis risk

## Abstract

Despite all the efforts to optimize the meniscus prosthesis system (geometry, material, and fixation type), the success of the prosthesis in clinical practice will depend on surgical factors such as intra-operative positioning of the prosthesis. In this study, the aim was therefore to assess the implications of positional changes of the medial meniscus prosthesis for knee biomechanics. A detailed validated finite element (FE) model of human intact and meniscal implanted knees was developed based on a series of in vitro experiments. Different non-anatomical prosthesis positions were applied in the FE model, and the biomechanical response during the gait stance phase compared with an anatomically positioned prosthesis, as well as meniscectomized and also the intact knee model. The results showed that an anatomical positioning of the medial meniscus prosthesis could better recover the intact knee biomechanics, while a non-anatomical positioning of the prosthesis to a limited extent alters the knee kinematics and articular contact pressure and increases the implantation failure risk. The outcomes indicate that a medial or anterior positioning of the meniscus prosthesis may be more forgiving than a posteriorly or laterally positioned prosthesis. The outcome of this study may provide a better insight into the possible consequences of meniscus prosthesis positioning errors for the patient and the prosthesis functionality.

**Electronic supplementary material:**

The online version of this article (10.1007/s11517-020-02158-0) contains supplementary material, which is available to authorized users.

**Figure:**
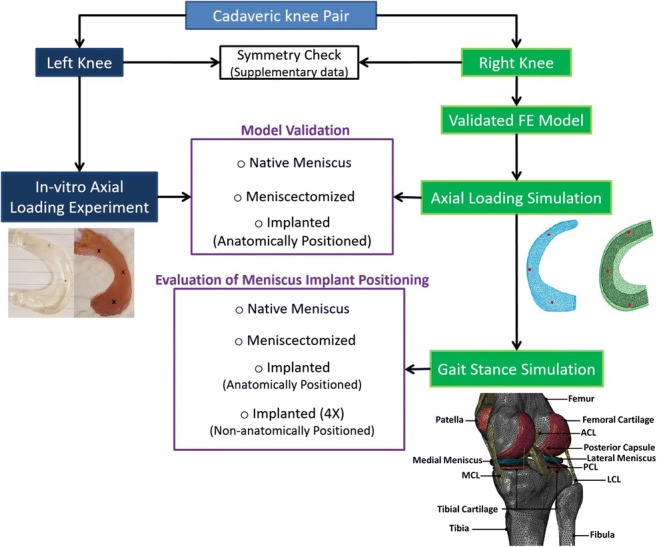
Graphical abstract

## Introduction

Medial meniscus injuries are among the most common knee-related injuries. When the medial meniscus cannot function properly due to severe damage or degeneration, it might be partially resected (partial meniscectomy). The more meniscus tissue is resected the higher the chance of osteoarthritis (OA) [1]. This increase of OA may lead to pain and functional impairment. When most of the meniscus is absent, replacement with a meniscal allograft may be an option. After transplantation the pain is reduced and patients typically have an improved quality of life [2]. However, problems related to the availability and sizing of allografts has driven the search for an alternative treatment [3–5]. An on-the-shelf meniscus prosthesis may overcome the shortcomings of meniscal allografts.

For a meniscus prosthesis, the geometry, material properties, fixation type, and prosthesis positioning are believed to be crucial factors [6–8], which need to be assessed thoroughly before clinical implementation. The influence of geometrical specifications of the medial meniscus prosthesis [9–11] and the material properties of the prosthesis [12–14] on the knee biomechanics have previously been studied, as have different meniscus prosthesis fixation types [15, 16].

In our lab, a novel anatomically shaped, polycarbonate urethane total meniscus prosthesis was recently developed. The prosthesis geometry was extracted using statistical shape modeling based on 35 subjects [17], and optimized implementing computational modeling [18]. The meniscus prosthesis consists of a stiff core embedded in a soft flexible (polymer) body. Based on cadaveric and animal experiments, proper materials for the meniscus prosthesis were selected [19–21]. The composite structure of the meniscus prosthesis allows for flexible articulations, while simultaneously constraining excessive prosthesis deformation. The prosthesis polymeric horns can pivot around metallic posts to minimize torque loads to the prosthesis horns [22]. Several studies have been performed to improve the geometry, material properties, and fixation technique of the meniscus prosthesis [17–21, 23].

In analogy with meniscus allograft transplantation, positioning of a meniscus prosthesis may influence the biomechanical behavior in the knee [24]. In clinical practice, the success of the prosthesis, therefore, will depend on surgical factors such as the intra-operative positioning of the prosthesis. Wajsfisz et al. introduced a new arthroscopic technique for meniscal transplantation [25]. With their technique, they could achieve a placement accuracy of about ± 2 mm in anterior-posterior and ± 4 mm in medial-lateral directions. However, the influence of the implantation offset on joint biomechanics was not reported in their study. Sekaran et al. assessed the impact of posterior attachment dislocation of autografts on the contact pressure on the medial tibia plateau in a cadaveric study [26]. Their results revealed an alteration in contact pressures in a simplified loading condition, when the posterior horn of the native meniscus was fixated posteriorly. While the influence of the shifted placement of an allograft has previously been investigated [25, 26], a study on the significance of accurate meniscus prosthesis positioning on knee joint biomechanics is still missing. Despite all the efforts in optimizing the medial meniscus prosthesis design in order to replicate the function of intact medial meniscus, there can be major differences in both geometries and material properties between the developed meniscus prostheses and a native meniscus. Therefore, the results of the allograft positioning studies may not be expanded for the meniscus prosthesis. The sensitivity of knee mechanics to the (mal-)positioning of the prosthesis is still unknown, but of interest for the surgeon and engineers to optimize their surgical techniques and instrumentations.

The aim of this study was therefore to assess the implications of positional changes of a medial meniscus prosthesis. The outcome of this study may provide a better insight into the possible consequences of meniscus prosthesis positioning errors for the patient and the prosthesis functionality. This study may also open a discussion for possible risks of OA due to the mechanical factors induced by implantation errors. It should be noted that as the computational modeling performed in this study was based on cadaveric experimental set-up (axial loading), a full direct model validation against experimental measurement was not always possible, and some of the simulations (gait stance) were outside the domain of direct validation.

## Methods

**Fig. 1:**
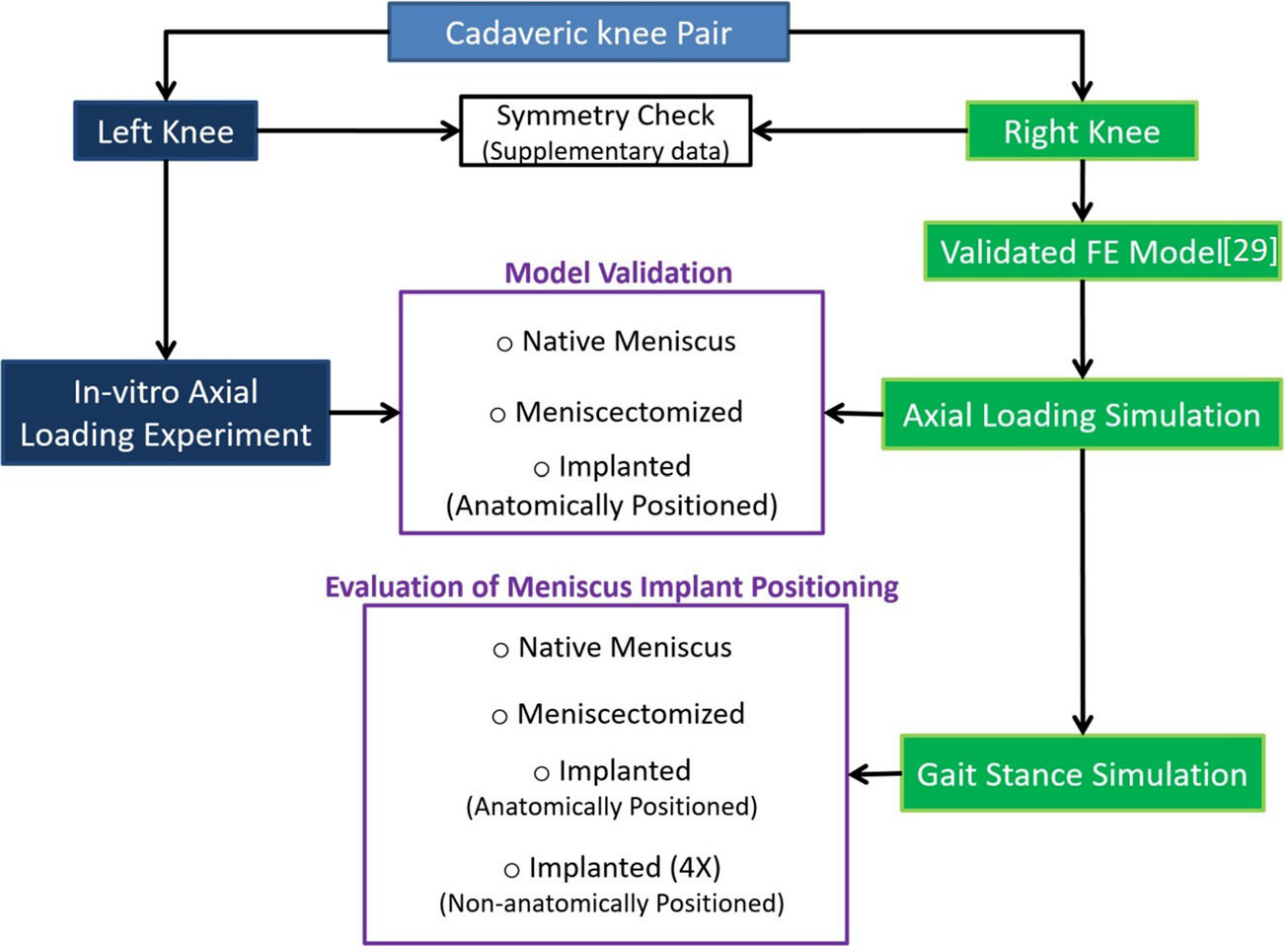
Schematic illustration of the workflow of the current study

A pair of fresh frozen cadaveric knees, with no sign of injury and surgery, was selected to follow the workflow of this study, as schematically illustrated in Fig. 1. The specimens were received from the Anatomy Department of Radboud University Medical Center with a permission statement for experimental use and all methods were carried out in accordance with the relevant guidelines and regulations for using cadaveric materials. Due to the time required for preparation and intensive experimentations for FE model development and implantation, which could highly affect the tissue quality, for this particular study, a symmetrical pair of knees was required.

After checking the symmetry of two knees (Supplementary Data), the right knee was used for experiments to generate data for developing a validated FE model (our earlier study [27]), and the left knee was used for a meniscus implantation study. The in vitro experiment on the left knee was then simulated with the validated FE model of the right knee, and the FE model predictions were further validated against experimental measurements. In addition to the anatomically positioned meniscus prosthesis, different non-anatomical prosthesis positioning was applied in the FE model. A stance gait cycle was simulated with the intact knee model, meniscectomized model, and anatomically positioned and non-anatomically positioned prostheses to assess the influence of different implantations on the biomechanics of the joint and prosthesis.

### In vitro axial loading experiment

**Fig. 2:**
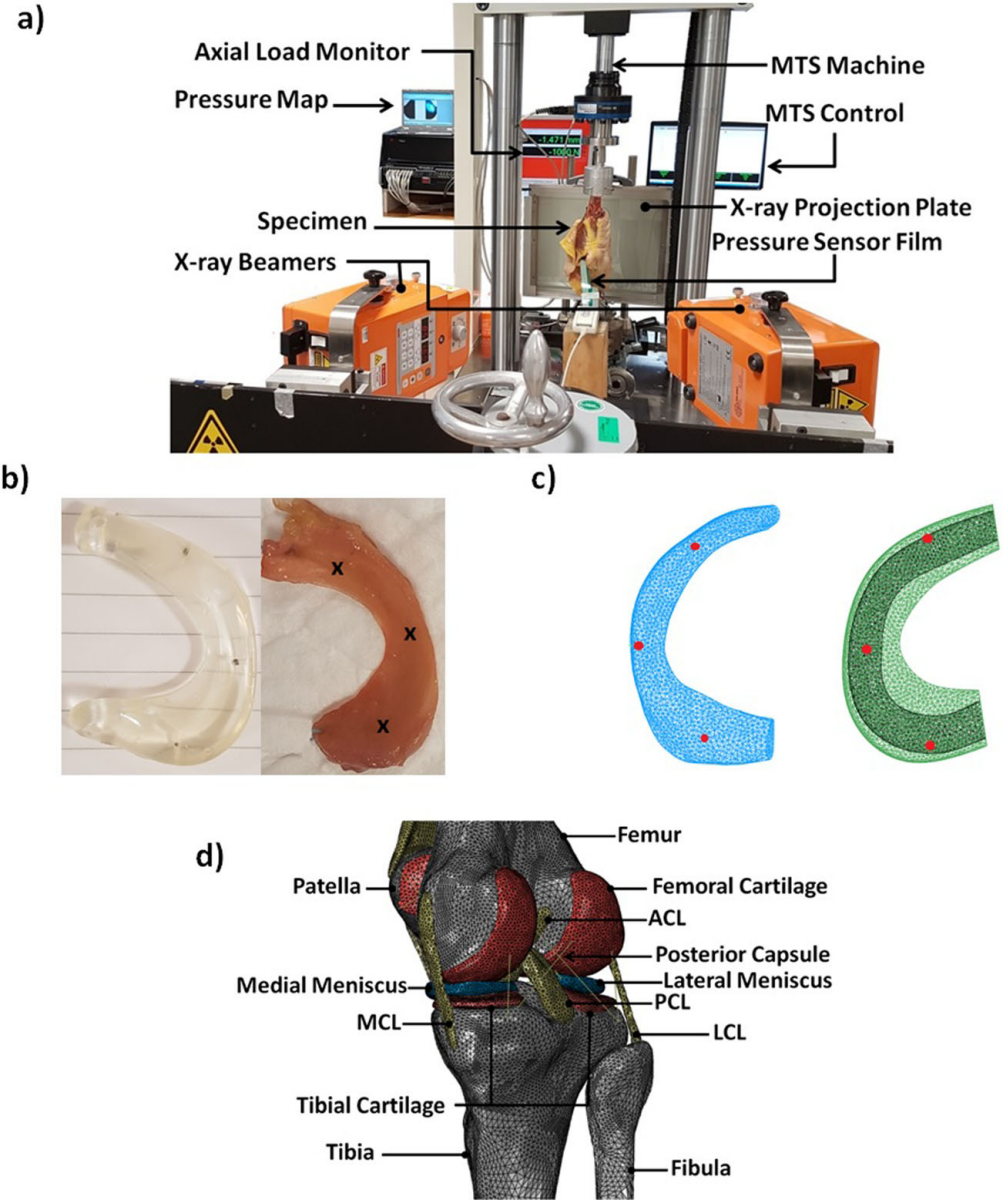
In vitro experimental setup (axial loading) to assess the biomechanical response of the cadaveric left knee (**a**), and the motion of the injected titanium beads could be quantified using RSA techniques in the native meniscus (the figure shows the isolated excised meniscus) and meniscal prosthesis (**b**) which were compared with the representative nodes (**c**) in the detailed validated FE model (**d**) of the contralateral knee (right knee)

The left knee was used for in vitro implantation experiments (Fig. 2a). After measuring the dimensions of the tibial plateau from a calibrated X-ray (medial-lateral (ML) width and anterior-posterior (AP) length), a proper sized meniscus prosthesis was selected. The size selection was based on the criteria described by Pollard et al. and Dienst et al. [28, 29] and the previous study on sizing of the meniscus prosthesis [18]. The ML width and AP length of the meniscus prosthesis, therefore, fitted with the corresponding dimensions in tibial plateau, with a proper size match (ML width > 80% and AP length > 90%). First, small tantalum markers (diameter 1 mm) were injected into the femur (three markers) and tibia (three markers). Next, the joint with the markers injected was CT-scanned in order to define the relative position of the markers with respect to the bony segment. Tantalum beads (diameter 0.5 mm) were also injected into the native meniscus (reaching through dissected capsule) and the meniscus prosthesis in the anterior, posterior, and middle regions (Fig. 2b). During the experiment, the positions of the markers were captured using Roentgen stereophotogrammetric analysis (RSA; Fig. 2a) and in-house developed scripts (MATLAB R2013a, Natick, MA).

The joint was prepared to be positioned in a mechanical testing machine (MTS, MTS Systems Corporation, Eden Prairie, MN, USA) in an extended position. A calibrated pressure sensitive film (Type 4011, Tekscan Inc., Boston, MA, USA) was inserted from the anterior side underneath the medial meniscus by an experienced knee surgeon. An axial load of 1000 N was applied to the femur, and the pressure map was recorded after 30 s of applying the load. The medial meniscus was removed by the surgeon to replicate the total medial meniscectomy, and the same loading condition was applied to the joint. Eventually, the meniscus prosthesis was inserted in the joint space using bone screw fixations at the centre of the anterior and posterior attachments of the excised native meniscus. The load was re-applied to the implanted knee while the contact pressure was recorded.

Based on the RSA techniques, the medial-lateral (ML) and anterior-posterior (AP) motions of the injected titanium beads were calculated using in-house developed MATLAB scripts as indications for the native meniscus and meniscus prosthesis deformation at different regions. Eventually, the implanted joint was CT-scanned after the experiment for an accurate prosthesis positioning in the FE model, following the fixation screw holes in tibia.

### Axial loading simulation (finite element modeling of in vitro experiment)

A detailed FE model of the right knee was developed in Abaqus v6.13 (Pawtucket, RI, USA) based on the laxity experiments. The FE model was subsequently validated based on validation tests against measured kinematics and contact pressure at tibiofemoral articular surfaces (Fig. 2d) [27, 30].

n the FE model, cartilage was modeled as non-linear Neo-Hookean hyperelastic isotropic, in which the strain energy function *ψ* is described as a function of the first invariant of the left Cauchy-Green deformation tensor (*I*_1_) and the elastic volume ratio (*J*):

1 $$ \psi ={C}_{10}\left({I}_1-3\right)+\frac{1}{2D}{\left(J-1\right)}^2 $$

In this equation, *C*_10_ and *D* are the Neo-Hookean constant and the inverse of the bulk modulus, respectively, which were calculated based on experimental compressive tests on 11 cadaveric knees [31] (*C*_10_=0.86 MPa and *D* = 0.048 MPa^−1^).

Menisci were modeled as transversely isotropic with circumferentially oriented fibers, implementing the Holzapfel-Gasser-Ogden (HGO) hyperelastic model [32]. The strain energy function *ψ* is described as a function of Neo-Hookean terms, representing the non-collagenous matrix, and ${\overline{I}}_{4\left(\alpha \alpha \right)}$, pseudo-invariants of $\overline{\mathrm{C}}$ and A_α_ (directions of the fibers in the reference configuration):

2 $$ \psi ={C}_{10}\left(\overline{I_1}-3\right)+\frac{1}{2D}\left(\frac{(J)^2-1}{2}-\mathit{\ln}\ (J)\right)+\frac{k_1}{2{k}_2}\left\{\exp \left[{k}_2{\left\langle {\overline{E}}_{\alpha}\right\rangle}^2\right]-1\right\} $$

with:

3 $$ {\overline{E}}_{\alpha }=\kappa \left(\overline{I_1}-3\right)+\left(1-3\kappa \right)\left({\overline{I}}_{4\left(\alpha \alpha \right)}-1\right) $$

Constants *k*_1_ and *k*_2_ are material parameters, and κ ($0\underset{\_}{<}\kappa \underset{\_}{<}\frac{1}{3}$) describes the level of dispersion in the fiber directions. When κ = 0, all fibers are perfectly aligned, and $\kappa =\frac{1}{3}$ describes an isotropic material [33]. The meniscus prosthesis materials (polycarbonate urethane, Bionates grade II 80A and 75D, DSM Biomedical, Geleen, The Netherlands) were modeled as isotropic neo-Hookean material for the prosthesis body (*C*_10_=1.93 MPa and *D* = 0.001 MPa^−1^) and linear elastic material (*E* = 71 MPa, ν = 0.48) for the stiff meniscus core, based on the material specifications.

The in vitro experimental condition was replicated in the FE model of the right knee, following the initial joint orientation measured using RSA. In order to validate the prediction of the FE model, the contact pressure and contact area at the medial tibia plateau were compared with the experimentally measured values in three cases (native, meniscectomy, implanted). Moreover, the motions of the native meniscus and the meniscal prosthesis were compared in the FE model (Fig. 2c).

### Gait stance simulation with different meniscus prosthesis positioning

To investigate the effect of prosthesis malpositiong, prosthesis was then positioned 2 mm anteriorly, 2 mm posteriorly, 4 mm laterally, and 4 mm medially, according to the reported positioning errors [25]. A full stance phase of straight walking cycle was simulated with the knee model with native meniscus (intact knee), the meniscectomized knee, the anatomically positioned prosthesis, and the four different shifted non-anatomical implantations (anterior, posterior, medial and lateral), with a dynamic explicit solver [34]. The loads were adjusted based on the normalized in vivo loads produced from eight subjects, in the Orthoload database [35], and the weight of the cadaveric subject, following the ASTM International standard guide (F3141-15) [36]. The tibia was fully constrained, and the loads and flexion were applied to femur, respectively in tibial and femoral frames (Supplementary Data). The knee kinematics (in knee coordinate system described by Grood and Suntay [37]), the displacement of the native meniscus and meniscal prosthesis, the contact variables at tibial plateau, and the force at the attachment of the meniscal prosthesis (reaction force at attachment points representing fixation screws) were compared to assess the influence of prosthesis positioning on the knee joint biomechanics.

## Results

### Model validation (in vitro experiment versus finite element simulation)

**Fig. 3:**
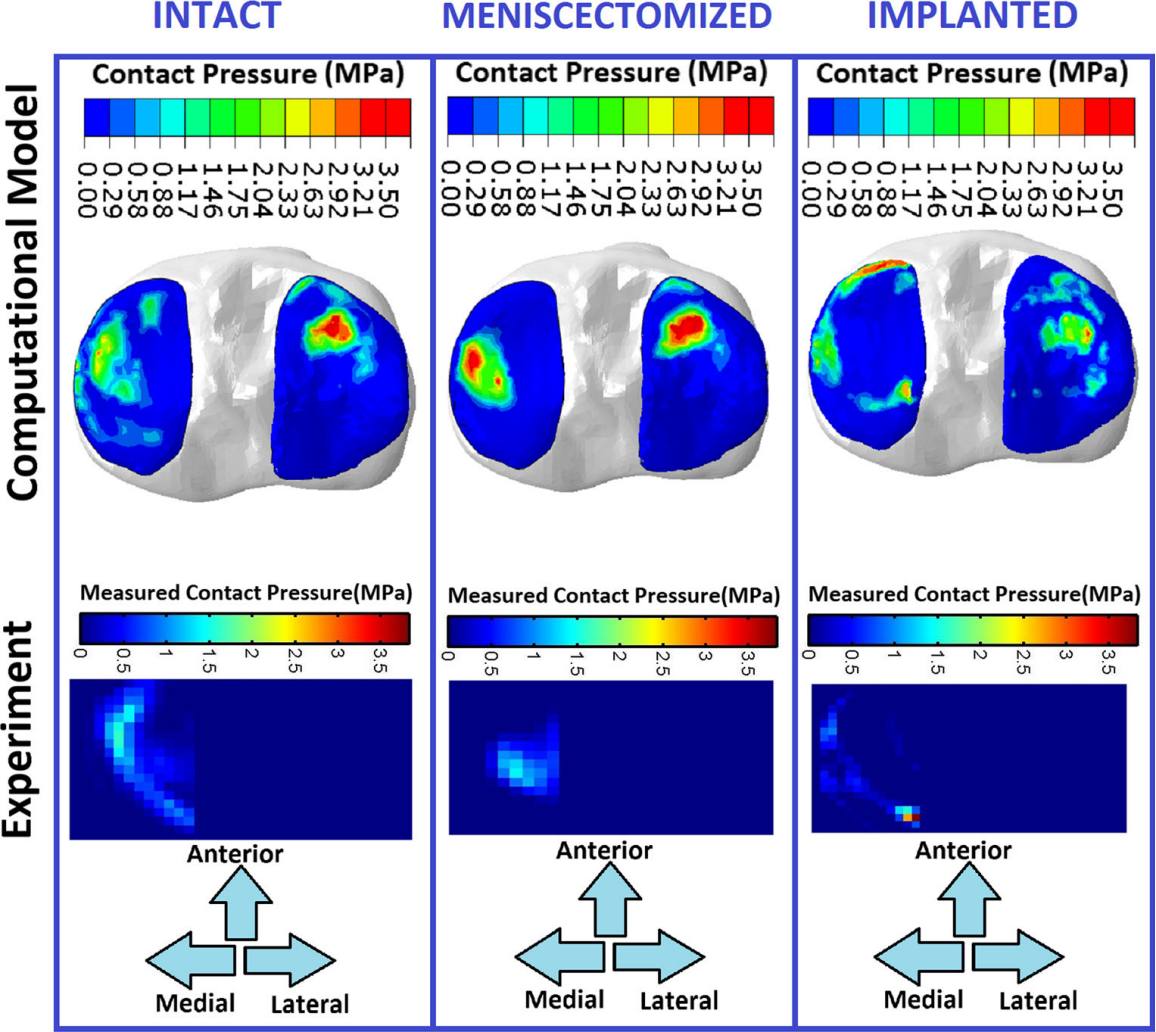
The contact pressure at tibial cartilage predicted by the FE model of the right knee (top) and measured during axial loading experiment (bottom) on the left knee, for the knees with native meniscus, total medial meniscectomy, and meniscus prosthesis. In order to facilitate the comparison, the experimental pressure maps were horizontally flipped

**Fig. 4:**
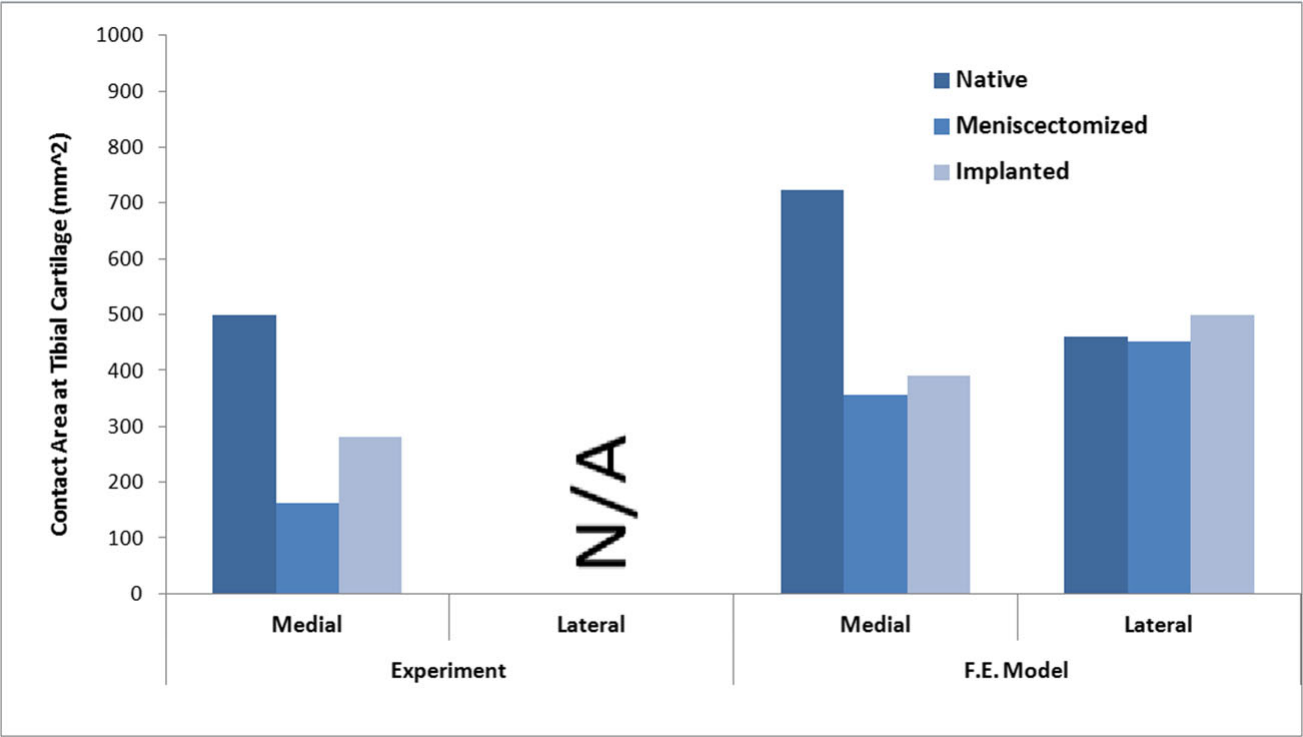
Articular contact area on tibial plateau (medial) during the axial loading experiment (left) and the calculated contact area in the FE model on both medial and lateral tibial plateaus in axial loading simulation

In the axial loading case, the computational (FE) model could predict the changes in the contact pressure pattern comparable to the experimental measurement at the medial tibial plateau (Fig. 3). As Fig. 4 illustrates, a similar trend was seen between the experimental measurement and computational prediction for contact area at the medial tibial cartilage.

The motion of the native meniscus and meniscal prosthesis under axial loading, as measured during the experiment and calculated in the FE model, is shown in Table 1. The FE model could predict the motions of the markers in both medial and anterior directions, with a reasonable agreement with experimental measurements, for both the native meniscus and the prosthesis.

**Table 1:** The medial and anterior displacements of the injected tantalum markers in native meniscus and meniscus prosthesis and in the FE model, under axial loading

		Medial displacement (mm)				Anterior displacement (mm)			
		Anterior marker	Middle marker	Posterior marker	Average	Anterior marker	Middle marker	Posterior marker	Average
Native meniscus	Experiment	1.02	1.40	0.61	1.01	− 0.89	− 1.30	− 0.86	− 1.02
	FE model	0.64	0.67	1.07	0.79	− 0.43	− 0.85	− 1.89	− 1.06
Meniscus prosthesis	Experiment	0.60	0.63	0.27	0.50	0.63	0.38	0.31	0.44
	FE model	0.49	1.66	0.39	0.85	2.80	1.85	0.48	1.71

### Evaluation of meniscus prosthesis positioning (computational outcomes)

#### Knee kinematics

**Fig. 5:**
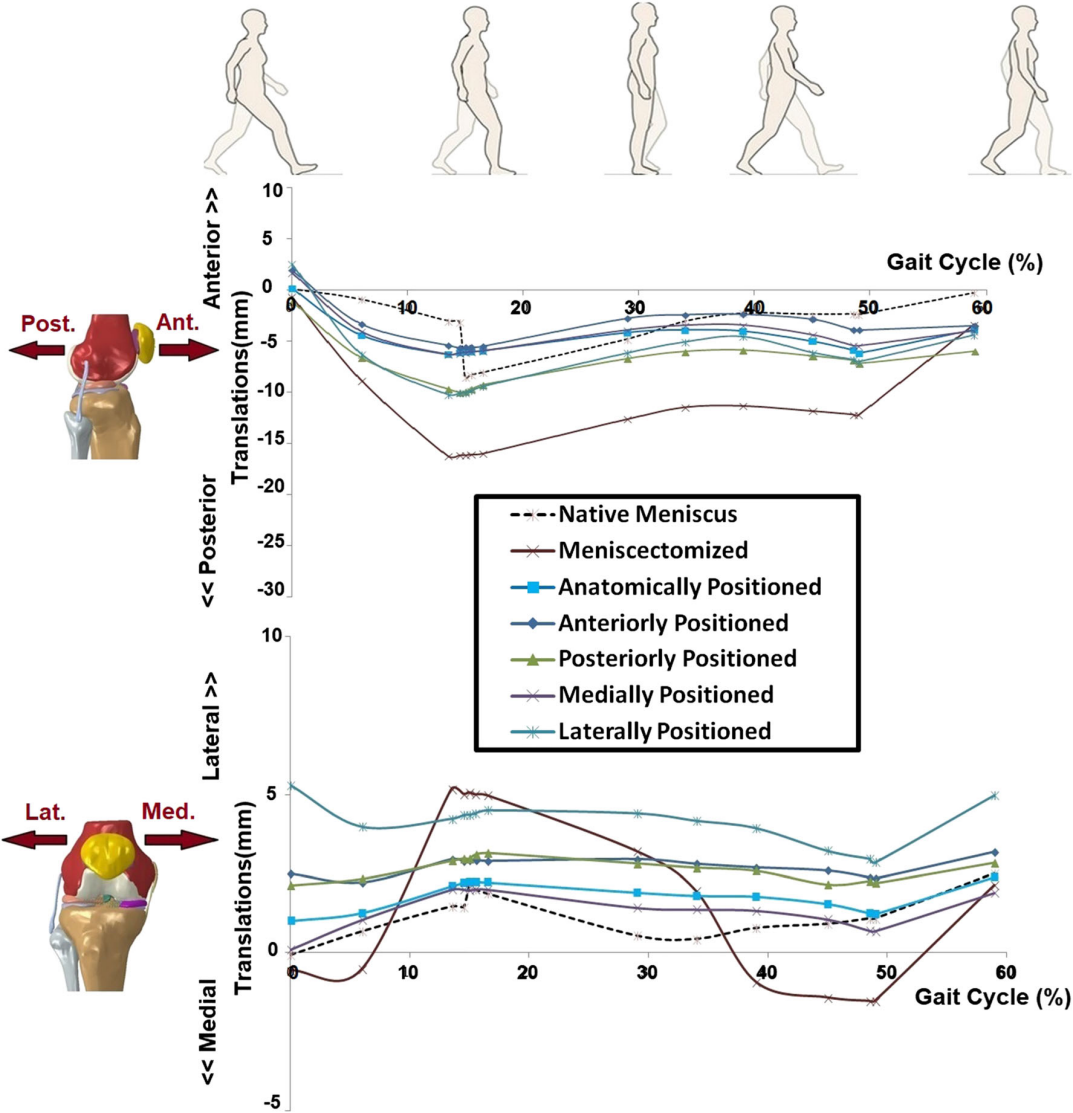
Translational (anterior-posterior and medial-lateral) kinematics of the knee joint during a complete gait stance phase simulation, for the knees with native meniscus, total meniscectomy, anatomically positioned meniscus prosthesis, and four non-anatomically (anteriorly, posteriorly, medially, and laterally) positioned meniscus prosthesis

**Fig. 6:**
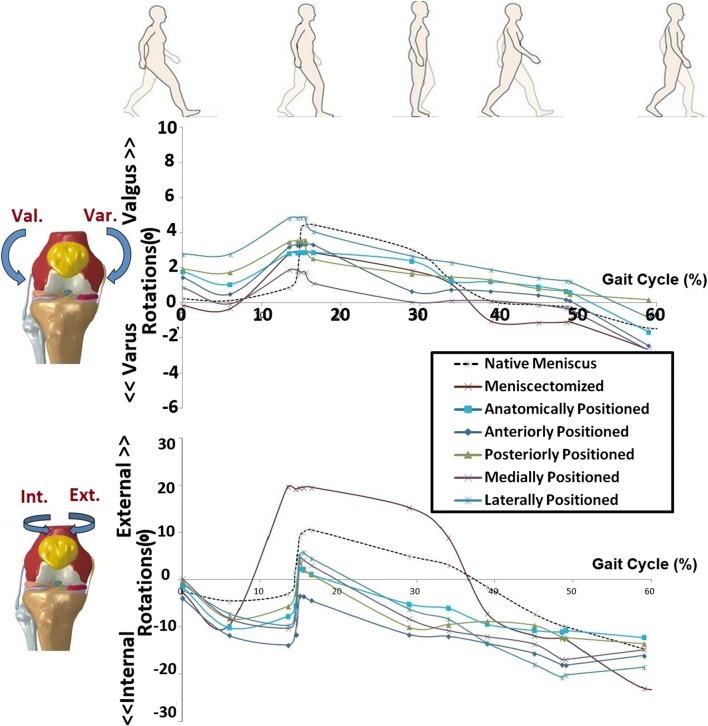
Rotational (valgus-varus and internal external) kinematics of the knee joint during a complete gait stance phase simulation, for the knees with native meniscus, total meniscectomy, anatomically positioned meniscus prosthesis, and four non-anatomically (anteriorly, posteriorly, medially, and laterally) positioned meniscus prosthesis

The FE model demonstrated that the meniscectomized knee joint had an increased medial-lateral translation (max. 4 mm) and anterior-posterior translation (max. 11 mm), both at the load acceptance phase (Fig. 5). Valgus rotation was reduced by meniscectomy, as shown in Fig. 6. Implantation at the anatomical position could partially recover the intact knee joint kinematics (Figs. 5 and 6). A non-anatomical positioned prosthesis influenced the anterior-posterior motions by less than 3.5 mm and the medial-lateral translations by less than 4 mm during the stance phase. A maximum alteration of 2° in valgus-varus and 6° in internal-external knee rotations was illustrated by non-anatomical positioning of the prosthesis.

#### Meniscal prosthesis motion

**Fig. 7:**
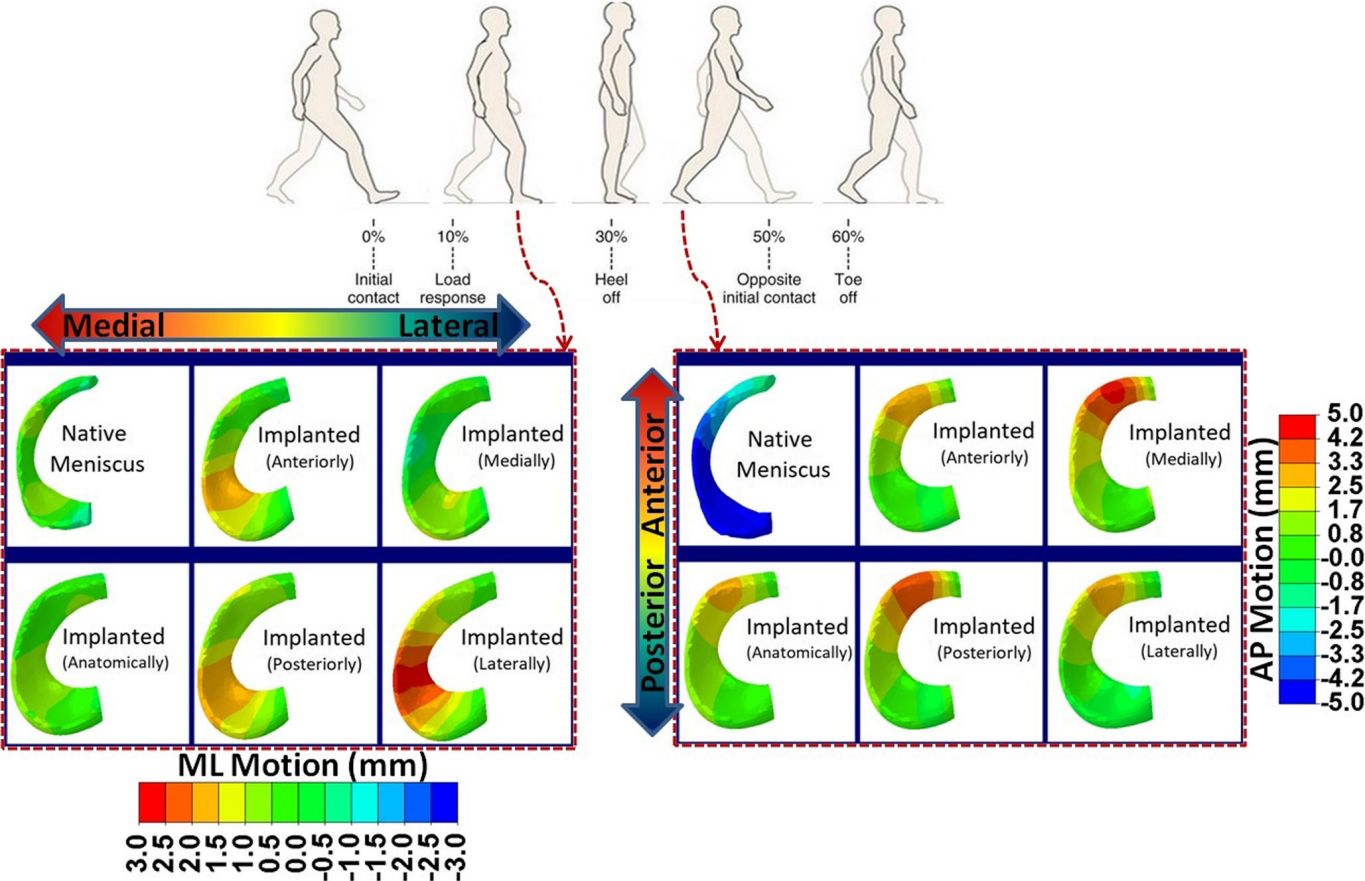
The displacements of native medial meniscus and medial meniscal prosthesis with the anatomical and four non-anatomical (anteriorly, posteriorly, medially, and laterally) positioning in medial-lateral direction (ML) at 20% and in anterior-posterior direction (AP) at 35% of a gait cycle

Comparing the meniscal prosthesis displacement in coronal plane (ML), the non-anatomical lateral positioning caused the largest prosthesis motion during the whole stance phase (Fig. 7). The posteriorly positioned prosthesis increased the displacement in the coronal plane, in the anterior region (30 to 60% of gait cycle) and posterior region (14 to 30% of gait cycle). The anteriorly positioned prosthesis resulted in a large motion in the coronal plane in the posterior region in the early stance (0 to 16% of gait cycle).

The medially positioned prosthesis showed the largest prosthesis motion in the sagittal plane (AP) at the end of the stance phase (30 to 50% of the gait cycle), maximally by ~ 4 mm (Fig. 7).

#### Contact variables

**Fig. 8:**
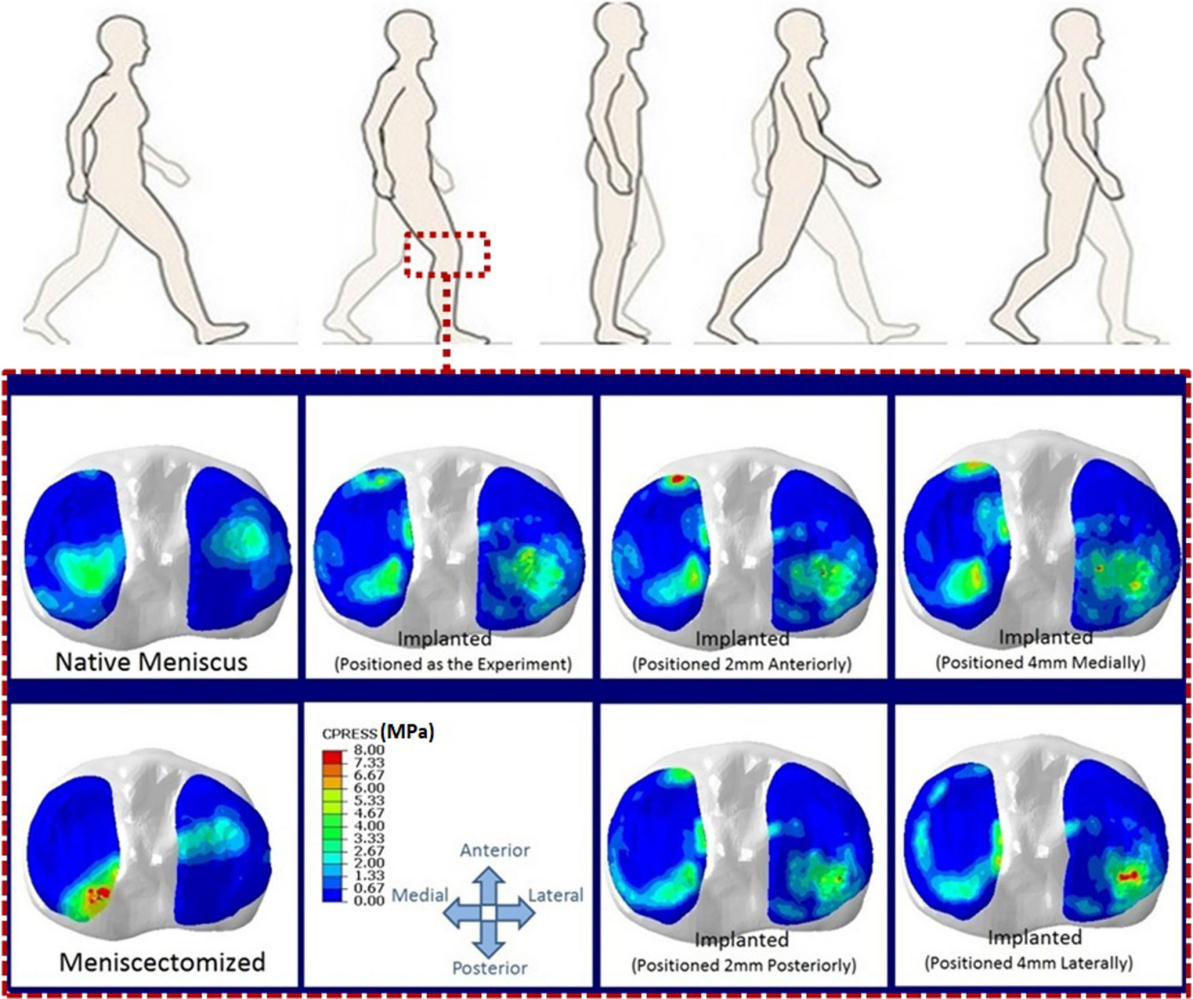
Contact pressure at tibial cartilages at loading response phase (20%) of a gait cycle for the knees with the native meniscus, total meniscectomy, anatomically positioned meniscus prosthesis, and four non-anatomical positioning of the meniscus prosthesis

In comparison with the intact knee, total meniscectomy increased the peak contact pressure at medial and lateral plateau, respectively, by 1.4 MPa and 0.3 MPa, during the stance phase simulation. With the anatomically positioned meniscal prosthesis, the peak contact pressure decreased with an average difference of 0.04 MPa (medial plateau) and 0.03 MPa (lateral plateau) relative to the intact knee. While the peak contact pressure was revealed to be less sensitive to an anterior or posterior prosthesis position, a lateral or medial position led to a slightly larger peak contact pressure, respectively, at both the lateral and medial plateaus (Fig. 8).

**Fig. 9:**
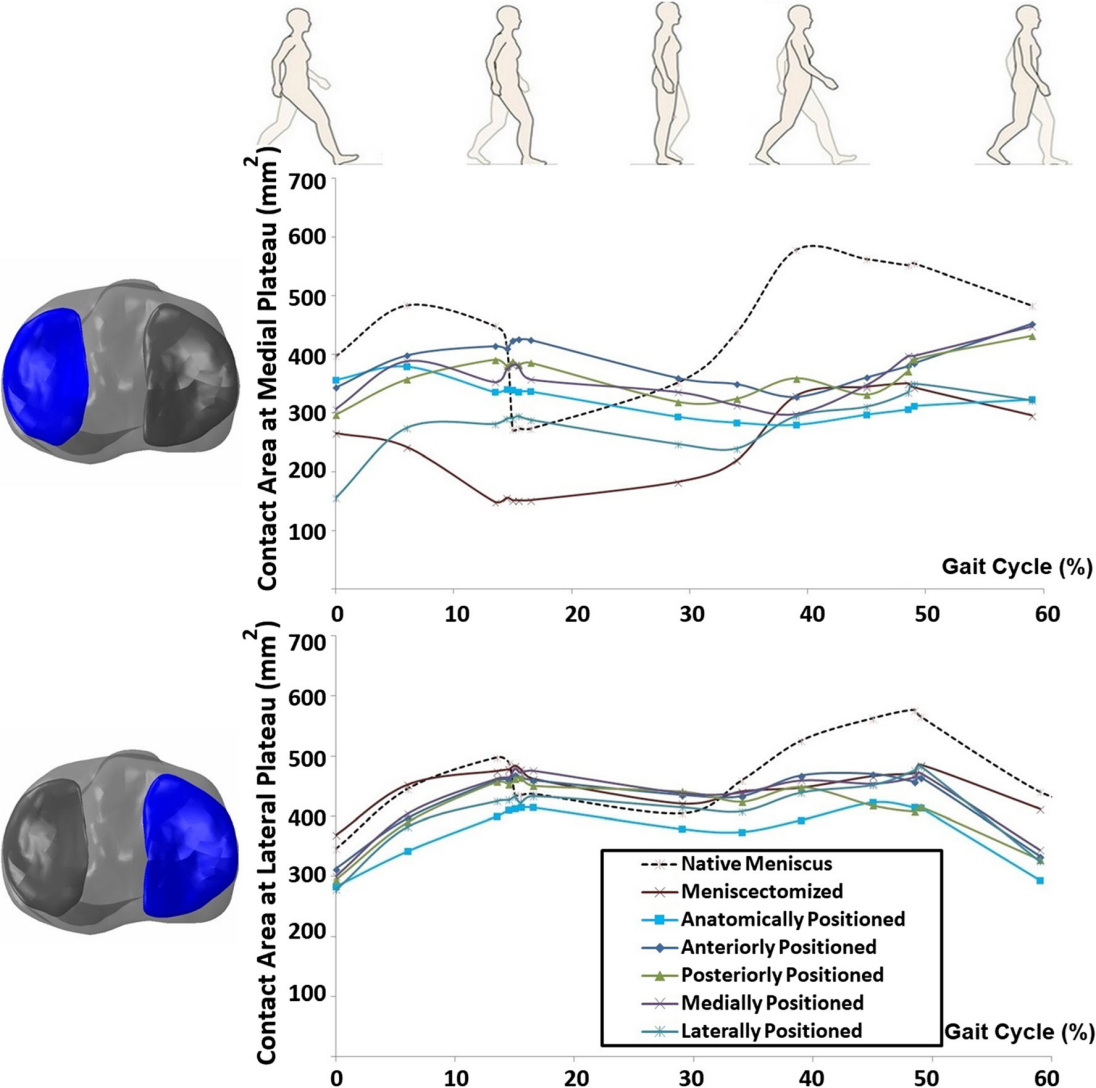
Articular contact area on tibial medial (top) and lateral (bottom) plateaus, during a complete gait stance simulation for the knees with native meniscus, total meniscectomy, anatomically positioned meniscus prosthesis, and four non-anatomically (anteriorly, posteriorly, medially, and laterally) positioned meniscus prosthesis

Meniscectomy predictably decreased the contact area at the affected plateau (medial plateau), while at the lateral plateau, the influence was negligible (Fig. 9). All the anatomical and non-anatomical implantation cases slightly increased the contact area at the medial plateau, although among the implantations the non-anatomical laterally positioned prosthesis showed the smallest contact area in this region.

#### Force at prosthesis horns

**Fig. 10:**
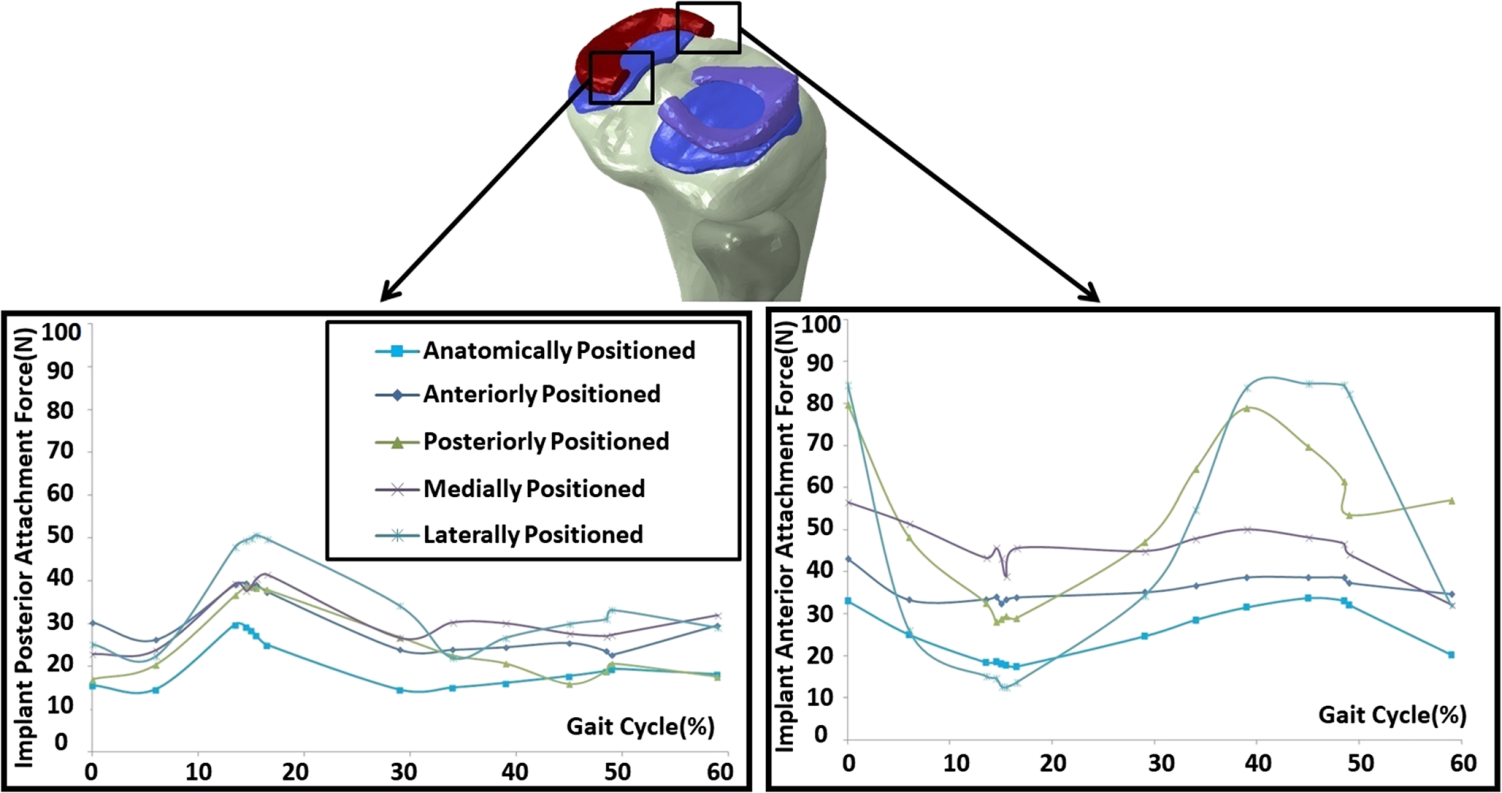
Total force at posterior (left) and anterior (right) fixations of meniscus prosthesis with anatomical and four non-anatomical positioning, during a gait stance simulation

In the laterally and posteriorly positioned implantation cases, the force at the anterior attachment of the prosthesis increased considerably in heel strike phase and also after the heel-off phase (30% of gait cycle; Fig. 10). Comparing with the anatomically positioned prosthesis, all the non-anatomically positioned prosthesis displayed a larger force at the posterior attachment, of which the laterally positioned prosthesis underwent the largest force.

## Discussion

In the current study, the influence of a non-anatomical positioning of a meniscus prosthesis on the knee biomechanics was assessed during a complete gait stance phase. For this purpose, the right knee of a symmetrical cadaveric pair was used to develop a validated FE model, while the left knee was utilized for an in vitro implantation experiment (axial loading) for further verification of the model outcomes validity (including the implanted knee model). Different non-anatomical prosthesis positions were applied in the FE model, and the mechanical response during the stance phase of gait compared with an anatomically positioned prosthesis, as well as with the intact and meniscectomized knee model. Although the FE model was validated against experimental tests with simple loads, due to the limitation in our testing apparatus, a full validation for more complicated loading condition was not possible. As a result, the FE model was partly utilized outside the domain of direct validation, and the results were compared with other studies as an indirect validation to check their sensibility.

The FE model was capable of predicting the motion of the native and meniscal prosthesis with an acceptable agreement with the in vitro experimental results. The simulated contact pressure and area at the tibial medial plateau were comparable with the experimental measurements. However, the contact areas measured during the experiments were smaller than those in the FE models, which may be due to limitations in the pressure sensitive films of covering the joint contact surface. It is worth mentioning, that for in vitro axial loading simulation the joint was constrained in valgus-varus direction to replicate the in vitro loading condition for validation purposes. It should therefore be noted that the outcomes of the in vitro loading simulation may not reflect the in vivo knee re-alignment conditions. Re-alignment after implantation was considered, however, for gait simulations.

The kinematic predictions of the FE model during the stance phase of gait for the intact knee agreed well with the literature in both knee translations and rotations [38–41]. The results of our gait simulation showed an increase in tibial internal and varus rotations and posterior motion due to total medial meniscectomy. This is in agreement with the findings of Netravali et al., in which similar changes were reported in 10 patients with medial meniscectomy compared with their healthy contralateral knees [42]. A sharp change appeared in posterior translation at 15% of gait cycle which coincided with the peak of quadriceps force [43], contributing to increasing the posterior force to femur [36]. Similar trend in femoral external rotation (15% of the gait cycle) was illustrated, where the femoral external torque was shown to be maximum in a gait cycle [36].

The outcomes of the simulation of the stance phase of gait showed that an anatomically positioned meniscal prosthesis could improve the knee joint biomechanics, although it could not fully recover the intact knee joint function. Non-anatomical positioning of the meniscal prosthesis could lead to a limited alteration in the joint kinematics. Werner et al. showed that contact distribution and contact loads on medial and tibial compartments significantly changed with a valgus-varus variation as little as 3° in gait, based on cadaveric experiments [44]. Similar findings of Engin et al. on human native knee joint confirm the high sensitivity of knee contact biomechanics to valgus-varus rotational configurations [45]. However, none of the non-anatomical prosthesis positionings led to a valgus-varus alteration beyond 2°, with respect to the anatomical positioning. The change in internal-external rotations, during the gait simulation by non-anatomical prosthesis positioning, can alter not only tibiofemoral joint behavior but also the biomechanics of patellofemoral joint. Patellar kinematics and patellofemoral contact pressure were shown to be slightly more sensitive to femoral internal rotation where an internal rotational change of 5° can alter the patellofemoral joint biomechanical behavior [46]. In anteriorly and laterally non-anatomical positioning cases, similar internal-external rotational change was reached. However, patellofemoral joint needs to be included in the model to assess the implications of the changes in tibiofemoral kinematics for patellofemoral mechanical responses. The alteration in the posteriorly directed joint behavior by non-anatomical posterior and lateral positioning can also lead to different cruciate ligament forces [47, 48].

In our study, we found that, in comparison with an anatomical prosthesis position, a non-anatomical position mostly resulted in a larger contact area at the medial tibial plateau. Sekaran et al. reported an increase in contact area at the medial plateau when an allograft is positioned medially to the anatomical location [26]. Their results illustrated that posterior positioning of the allograft leads to an even further increase in contact area on medial plateau. Our results showed a similar trend in contact area when the meniscus prosthesis was positioned more medial and posterior to the anatomical position. They also reported a posterior shift in the centroid of contact area (on tibial plateau) when the allograft was positioned posteriorly. This could be an indication of an increase in posterior translation of femur, similar to what our results showed for a non-anatomical posteriorly positioned meniscus prosthesis.

Despite the differences in strain behavior of meniscus prosthesis in different positioning, none of them reached beyond the failure strain of the materials implemented in the prosthesis. When the meniscus prosthesis was fixated more laterally or posteriorly, the force at the fixations changed dramatically in both magnitude and trend. This may increase the risk of prosthesis loosening at fixations and more particularly at the anterior fixation where the force fluctuation is larger [49–51]. The calculated forces at the fixations can be transferred to an isolated bone-screw FE model with a more realistic representation of the bone [52], in order to assess the risk of aseptic loosening due to fatigue failure [50].

The large prosthesis motion in the coronal plane due to the lateral positioning of the prosthesis may have an influence on shear stress at tibial cartilage, which can be correlated to the risk of OA progression [53]. A more detailed study on the cartilage stresses, however, requires a more sophisticated cartilage material model than the one used in this study. For instance, Wilson et al. demonstrated that local stress and strain behavior of the cartilage depends on local architecture of the collagen network [54]. Using a multi-scale FE modeling approach, the influence of prosthesis sliding motion on shear stress at medial tibial cartilage can be assessed. For this purpose, the resultant prosthesis motion from the current study can be applied to a simpler FE model with a more detailed representation for tibial cartilage.

There were several limitations in the current study. First, the computational FE model was developed and validated against an in vitro experiment on cadaveric specimen, while an in vivo model may give a more realistic insight into actual joint kinematics. However, due to the invasiveness of the measurements (contact pressure measurement, laxity measurement, RSA measurements, CT scanning), a cadaveric specimen-based computational modeling was unavoidable. Therefore, a cadaveric specimen-based detailed FE model was used which was intensively validated against in vitro experiments in our earlier study [30]. Second, the bones were modeled as rigid bodies, which were shown to be an acceptable assumption when contact variables are of interest. A more realistic inhomogeneous modeling of bones could enrich the model with more details of the screw-bone interface. Third, the boundary conditions for the simulation of gait were assumed to be similar for all cases, whereas in the meniscectomized case, the gait pattern might be different due to the lack of the meniscus or due to pain. However, this model was force-controlled, meaning that loads were applied to the knee joint while allowing for free joint adjustment during gait. As a result, and in contrast with motion-controlled models, similar loading for different cases might acceptably be applied. Considering the limitations in the pressure sensitive film (Tekscan4011; maximum pressure 3.45 MPa), an axial load of 1000 N was applied to further validate the FE outcomes for the purposes of this study which was similar to previous studies to assess meniscus performance (e.g., [23, 55, 56]). Larger axial loads representing actual in vivo loads can ensure FE outcomes validity for simulating daily-life activities (e.g., walking). And finally, due to uncertainties introduced mainly by not using the same knee for FE modeling and implantation experiment, the exact values in quantitative results may be used with care. In this comparative study, the results were, therefore, assessed to gain a general understanding on the implications of mal-positioning of prosthesis on knee behavior.

Despite the variations in the prosthesis mechanical properties and geometry, from the native meniscus, the anatomical placement of the meniscus prosthesis could better restore the intact knee biomechanics, comparing with all non-anatomical prosthesis positioning. Considering the morphological and structural (i.e., mechanical properties) differences between intact meniscus and the prosthesis, an optimal subject-specific meniscus prosthesis positioning (rather than an anatomical) may improve the implantation outcomes furthermore. To achieve this, the developed FE model in the current study could be combined with optimization algorithms, in order to optimize the meniscus prosthesis position in the injured knee based on the intact knee joint (contralateral knee) biomechanical outcomes. Although normally, a symmetrical behavior of contralateral knees is expected [57], the symmetry of two knees can be checked by comparing the geometrical dimensions of the contralateral knee joint. Subsequently, the calculated optimal meniscus prosthesis placement can be applied, for instance, using 3D printed surgical guides.

## Conclusion

This study showed that an anatomical positioning of the medial meniscus prosthesis could better recover the intact knee biomechanics, while a non-anatomical positioning of the meniscus prosthesis to a limited extent alters the knee kinematics and increases the risk of implantation failure. Our results indicate that a medial or anterior positioning of the meniscus prosthesis may be more forgiving than a posteriorly or laterally positioned prosthesis. As a result, if anatomical positioning of the prosthesis is not possible, for instance due to the mismatch in prosthesis horns distance and native meniscus attachment footprints, the meniscus prosthesis is recommended to be placed more to anterior or lateral sides.

## Electronic supplementary material

ESM 1 (DOCX 340 kb)
