# Supplementary material for: The implications of non-anatomical positioning of a meniscus prosthesis on predicted human knee joint biomechanics
Source: Med Biol Eng Comput. 2020 Apr 11;58(6):1341–55. doi: 10.1007/s11517-020-02158-0 (PMC7211793; doi:10.1007/s11517-020-02158-0)
Supplement: Supplementary file 1 — (DOCX 340 kb) [file 11517_2020_2158_MOESM1_ESM.docx]

**Supplementary Data**

**The Implications of Non-Anatomical Positioning of a Meniscus Prosthesis for Human Knee Joint Biomechanics**

Hamid Naghibi, Dennis Janssen, Ton van den Boogaard, Tony van Tienen, Nico Verdonschot

**1. Symmetry and laxity experiment**

In order to check the geometrical symmetry of the contralateral knees, the knees were scanned with proton density sequence, in a 3T Philips Ingenia MRI scanner (Philips Healthcare, Best, The Netherlands), with a slice thickness of 0.5mm. The knees were segmented using Mimics v18.0 (Materialise, Leuven, Belgium) to determine the bones and menisci. The bones geometry was then corrected based on the segmentation from CT data (Toshiba Aquilion ONE, Otawara, Japan). Total width of the medial meniscus (AP) and the width of the femur and tibial plateau were compared between the right and left knee.

To check the similarity in knee laxity, the left and right knee were prepared following a standard protocol and tested in a knee testing apparatus that allows for six degree of freedom motions [16-18]. Flexion-extension was applied to the femur, whereas the valgus-varus and internal-external rotations and anterior-posterior and medial-lateral translations were applied to the tibia. A series of laxity tests was applied to the knees while the position of the bony segments was recorded by an electromagnetic tracking system (3Space Fastrak, Polhemus Incorporated, VT, USA). In-house developed scripts (MATLAB R2013a, Natick, MA) were used to calculate the knee joint centre (similar to [1]), and to convert the raw tracking data to kinematics in the knee joint coordinate system [2], as described by Grood and Suntay [3].

Six different loading conditions were applied, at four different flexion angles (0, 30, 60 and 90°): an internal and external torque of 5.2 Nm, a varus and valgus moment of 12 Nm, and an anterior and posterior load of 100 N. These loads were based on literature values and provided sufficient laxity motion to characterize the knee ligaments without damaging the cadaveric specimens [4]–[7]. Each of the loading conditions was repeated three times to check the repeatability of the measurements. The joint laxity of the right and left knees were compared for the six loading regimes.

- Symmetry results

The dimensional comparison of the contralateral knees confirmed their geometrical symmetry in, for instance, femoral epicondylar width (~90 mm), tibial plateau width (~80 mm) and AP distance of medial meniscus horns (~ 50 mm). The knees showed comparable laxity in all directions (Figure 12-a), with a maximum difference of 1.8 mm in anterior and 2.1 mm in posterior translation (Figure 12-b), and 1.7° in valgus, 1.2° in varus (Figure 12-c), 2.7° in internal and 3.3° in external rotation (Figure 12-d).

**2. Boundary and loading conditions in gait stance simulations**

The loads were adjusted based on the normalized *in vivo* loads produced from eight subjects, in the Orthoload database [8], and the weight of the cadaveric subject, following the ASTM International standard guide (F3141-15) [9]. The tibia was fully constrained, and the loads and flexion were applied to femur, respectively in tibial and femoral frames.


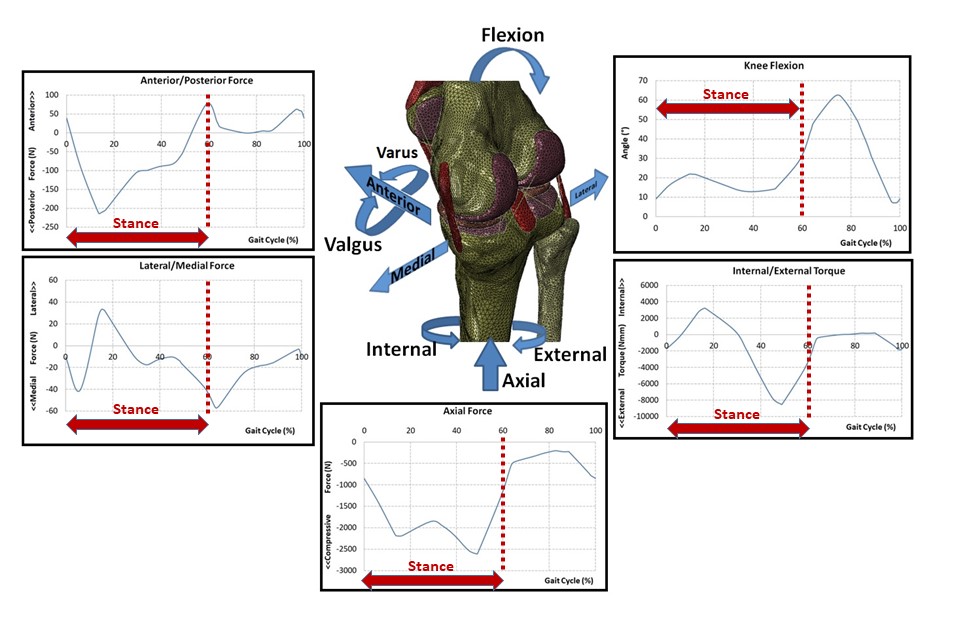


**Figure 11:** The loads and boundary conditions applied to the FE models to simulate gait stance phase.

| **a)**  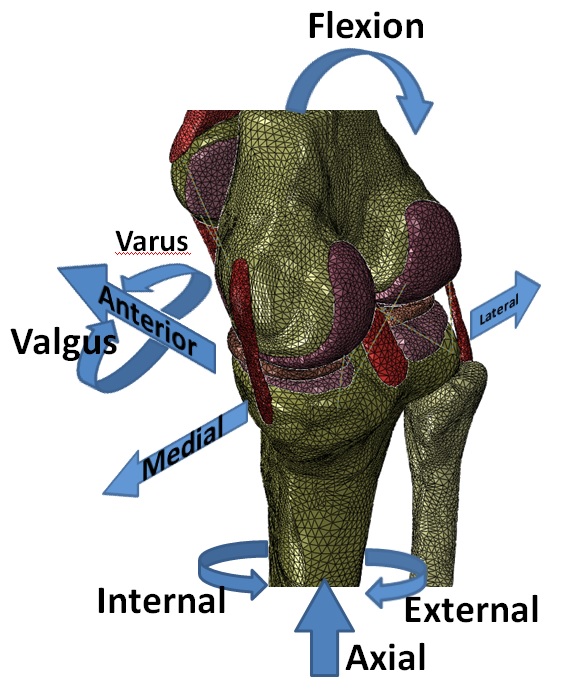 | **b)**  **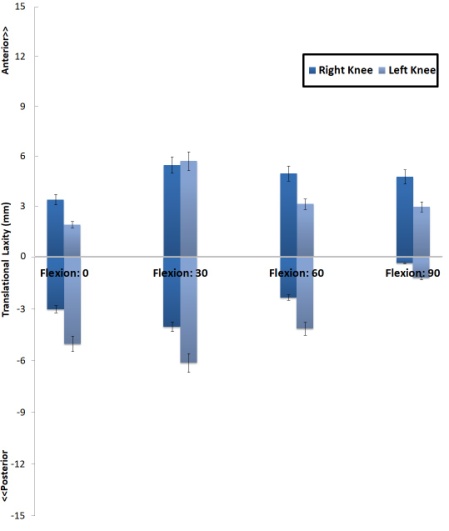** |
| --- | --- |
| **c)**  **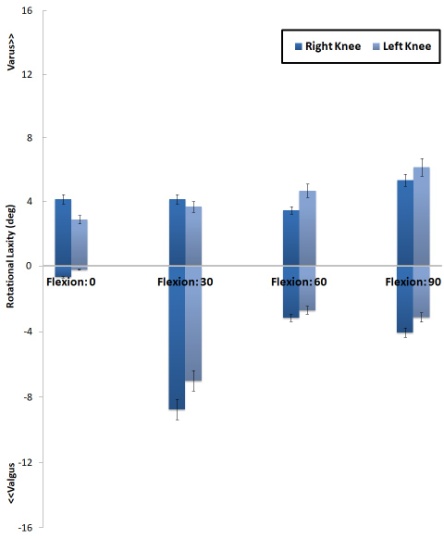** | **d)**  **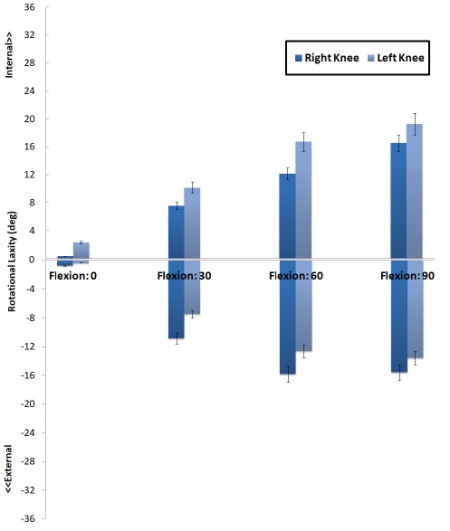** |

**Figure 12:** The results of the laxity comparison between the cadaveric right and left knee specimens in order to check their symmetry: a) different knee laxities indication in the FE model; b) anterior/posterior laxities against an anterior/posterior load of 100N; c) Valgus/varus rotational laxities while a valgus/varus moment of 12 N.m was applied; and d) Internal/external rotational laxities against an internal/external torque of 5.2 N.m.

**References**

[1] D. L. Miranda, M. J. Rainbow, E. L. Leventhal, J. J. Crisco, and B. C. Fleming, “Automatic determination of anatomical coordinate systems for three- dimensional bone models of the isolated human knee,” *J. Biomchanics*, vol. 43, no. 8, pp. 1623–1626, 2010.

[2] W. J. Zevenbergen, “Improved Anatomical Coordinate System of the Distal Femur Based on 3D Bone Geometry & Evaluation of the Inter- and Intra- Observer Variability of the Knee Ligament,” 2012.

[3] W. J. Grood, E.S.; Suntay, “A joint coordinate system for the clinical description of three-dimensional motions applications to the knee,” *J. Biomech. Eng.*, vol. 105, no. 2, pp. 136–144, 1983.

[4] D. L. Gollehon, P. A. Torzilli, and R. F. Warren, “The role of the posterolateral and cruciate ligaments in the stability of the human knee. A biomechanical study.,” *J. Bone Joint Surg. Am.*, vol. 69, no. 2, pp. 233–42, 1987.

[5] W. P. Seering, R. L. Piziali, D. A. Nagel, and D. J. Schurman, “The function of the primary ligaments of the knee in varus-valgus and axial rotation,” *J. Biomech.*, vol. 13, no. 9, pp. 785–794, 1980.

[6] Keith L. Markolf, J. S. Mensch, and Harlan C. Amstutz, “Stiffness and laxity of the knee - the contributions of the supporting structures,” *J. bone Jt. Surg.*, vol. 58, no. 5, 1976.

[7] M. a. Baldwin, C. W. Clary, C. K. Fitzpatrick, J. S. Deacy, L. P. Maletsky, and P. J. Rullkoetter, “Dynamic finite element knee simulation for evaluation of knee replacement mechanics,” *J. Biomech.*, vol. 45, no. 3, pp. 474–483, 2012.

[8] A. Rohlmann *et al.*, “Standardized Loads Acting in Knee Implants,” vol. 9, no. 1, 2014.

[9] *ASTM F3141-15, Standard Guide for Total Knee Replacement Loading Profiles*. West Conshohocken, PA: ASTM International, 2016.
